# Phytochemical analysis and Evaluation of hepatoprotective effect of *Maytenus royleanus* leaves extract against anti-tuberculosis drug induced liver injury in mice

**DOI:** 10.1186/s12944-020-01231-9

**Published:** 2020-03-16

**Authors:** Maria Shabbir, Tayyaba Afsar, Suhail Razak, Ali Almajwal, Muhammad Rashid Khan

**Affiliations:** 1 Atta-ur-Rahman School of Applied Biosciences, NUST, Islamabad, Pakistan; 2 Department of Biochemistry, Faculty of Biological Sciences, Quaid-i-Azam University, Islamabad, 45320 Pakistan; 3 Department of Community Health Sciences, College of Applied Medical Sciences, King Saud University, Riyadh, Kingdom of Saudi Arabia

**Keywords:** HPLC, Liver function tests, DNA damages, Histopathology, Antioxidant, Lipid peroxidation

## Abstract

**Background:**

Myrin®-p Forte is an anti-tuberclosis agent that can cause hepatic injuries in clinical settings. *Maytenus royleanus* (Celastraceae) is a medicinal plant, possesses antioxidant and anticancer activities. The hepatoprotective effect of the methanol extract of *Maytenus royleanus* leaves (MEM) against Myrin®-p Forte induced hepatotoxicity in mice was investigated.

**Methods:**

Mice were randomly parted into six groups (*n* = 6). Fixed-dose combination of Myrin®-p Forte (13.5 mg/kg Rifampicin, 6.75 mg/kg Isoniazid, 36.0 mg/kg Pyrazinamide and 24.8 mg/kg Ethambutol; RIPE] was administered for 15 days to induce liver injury. In treatment groups *MEM* (200 mg/kg and 400 mg/kg doses) and Vitamin B6 (180mg/kg) were administered prior to RIPE. Control group received 2% DMSO. Serum liver function tests, DNA damage, tissue antioxidant enzymes and histopathological alterations were studied. HPLC analysis was performed to determine the chemical composition using standard compounds.

**Results:**

The quercitin, gallic acid, luteolin, viteixin, apigenin, kaempherol, hyperoside and myricetin contents of all samples were determined by reverse-phase HPLC. Quercetin (0.217 mg/g dry weight) and luteolin (0.141 mg/g dry weight) were the major flavonoids identified in MEM. Myrin®-p Forte markedly (*p* < 0.05) deteriorated lipid profile and upregulated the concentration of LDH, AST, ALP, ALT and γ-GT in serum along with DNA fragmentation (37.13 ± 0.47%) and histopathological injuries in hepatic tissues of mice compared with the control group. Myrin®-p Forte increased (*p* < 0.001) lipid peroxidation and H_2_O_2_ while decreased (p < 0.001) the activity level of CAT, SOD, POD, GPx, GST, GSR, γ-GT and GSH. Co-administration of MEM (200 mg/kg; 400 mg/kg) or the vitamin B6 (180 mg/kg) to Myrin®-p Forte administered mice significantly ameliorated LDL, cholesterol, HDL and triglyceride content. Furthermore, MEM dose dependently corrected serum liver function tests, decrease % DNA fragmentation (17.82 ± 0.35 and 7.21 ± 0.32 respectively), DNA damage. MEM treated protect RIPE induced oxidative damage by enhancing antioxidants to oxidants balance. Histological examination comprehends biochemical findings.

**Conclusion:**

The antioxidant effects of MEM exerted the hepatoprotective potential against the Myrin®-p Forte induced hepatotoxicity in mice.

## Background

Tuberculosis (TB) a curable respiratory ailment instigated by *Mycobacterium tuberculosis*; mostly affecting the poor countries of Africa and Southeast Asia. According to World Health Organization (WHO), its prevalence recorded was 14 million, while 2.38 million deaths were estimated. Fixed dose combination of Myrin®-p Forte [contains Rifampicin (13.5 mg/kg), Isoniazid (6.75 mg/kg), Pyrazinamide (36.0 mg/kg) and Ethambutol (24.8 mg/kg); RIPE]. RIPE has been recommended by WHO for the intensive phase (2 months) followed by continuous treatment of Rifampicin and Isoniazid for 4–6 months [1]. However, this regimen causes hepatic injuries in clinical settings [2]. The clinical symptoms of anti-TB drug appear in nonspecific elevation of transaminases to fulminant of liver failure [3]. Hepatic injuries have been induced with isoniazid and rifampicin in experimental animals [4]. It is suggested that isoniazid is metabolized into monoacetyl hydrazine as well as isonicotinic acid; the latter can be activated through metabolic oxidation of cytochrome P-450 to toxic species causing hepatic damages. Rifampicin aggravated the hepatotoxicity due to its high amidase activity and is involved in release of large concentrations of acetyl-hydrazine from isoniazid [5]. The reactive metabolites of an acetyl-hydrazine bind with hepatic proteins causing injuries [6]. Likewise the pyrazinamide is metabolized by the hepatic xanthine oxidase as well as microsomal amidase and the intermediaries; pyrazinoic acid and 5-hydroxy pyrazinoic acid are considered to be involved in hepatotoxicity [7]. Although the exact mechanism and contributing factors of hepatotoxicity induced with anti-tuberculosis drug are not clear; reactive oxygen species (ROS)-mediated oxidative damage is postulated to be the main factor of lipid peroxidation and consequently the hepatic injuries. Alterations in the enzymatic and non-enzymatic entities of the cellular defence mechanism have been reported with the use of anti-TB drug [8]. Hydrazine declines the level of cellular glutathione (GSH) and suggested to minimize the oxidative defence mechanism and consequently cause cellular injuries and death [9].

The plants provide a natural source to treat various aspects of diseases. It has been observed that most of the plant based drugs impart their therapeutic potential by exhibiting antioxidant activities. The plant extracts comprise a range of compounds including alkaloids, glycosides, flavonoids, fatty acids, saponins, sterols, and others. Polyphenolic compounds of plants are of remarkable importance because they confer such hydroxyl groups that show scavenging potential for free radicals [10]. On account of potent antioxidant properties; during current years many species of plants have been evaluated for the management and treatment of various ailments. For this reason, research work is being conducted to suggest an approach that involves certain agents tending to alleviate the anti-TB drug induced hepatotoxicity. *Maytenus royleanus* belonging to the family Celastraceae is distributed in the lower Himalayas surrounding Islamabad and on dry sunny slopes of Kaghan (KPK) Pakistan. Its bark is used by the local population in gastrointestinal disorders [11]. In our previous investigations, we have identified caffeic acid, quercetin 3-rhamnoside, triterpenoids by HPLC, flavonoids and tannins by quantitative phytochemical analysis, while LC-MS fingerprinting revealed Anthocyanines, Phenolics, Chlorophylls, Macro and micro constituents in MEM [10, 12]. In vitro antioxidant and anti-lipid peroxidation activities of *M. royleanus* leaves have been recorded in our previous studies [13]. Furthermore, we have observed significant decrease in prostate cancer cell viability and clonogenic survival with the methanol extract of *M. royleanus* leaves. These findings are associated with a significant inhibition in tumor growth and decline in serum level of prostate-specific antigen (PSA) in athymic nude mice [12]. Many species of *Maytenus* were explored for their antioxidant and hepato-protective potential in in vivo system. The methanolic extract from *Maytenus robusta* leaves showed hepatoprotective effect in mice and HepG2 cells against CCL4 induced toxicity. Extract reduced the hepatic histological damages and normalize hepatic biomarkerss. The antioxidant effect of *M. robusta* in liver tissue promoted the reduction in lipoperoxides levels increased the reduced glutathione content and increased the activity of superoxide dismutase, catalase, and glutathione-S-transferase Moreover, the extract reduced hepatic inflammation by diminishing myeloperoxidase activity, TNF and interleukin-6 levels by its antioxidant effects [14]. Similarly, *Maytenus emarginata* ethanol extract possess potent hepatoprotective effect. Treatment of rats with the *Maytenus emarginata* extract showed marked decrease in levels of serum ALP, ALT as well as AST with elevation of serum protein and albumin [15].

Based on the hepatoprotective potential of related species in animal models, and diversified in vitro *and* in vivo pharmacological properties of *M. royleanus,* the current experiment was designed to evaluate the protective effects of methanol extract of *M. royleanus* leaves against the anti-TB drug induced hepatotoxicity in mice. The chemical composition was analysed by HPLC using standard flavonoid compounds. Various biochemical parameters, lipid profile, DNA ladder assay and histological investigations were done to apprehend the protective impact of plant extract against anti-TB drug induced hepatotoxicity.

## Methods

### Plant collection

Leaves of *M. royleanus* were collected in March 2011 from village Lehtrar, Tehsil Kotli Sattian, District Rawalpindi, Pakistan. The plant identity was verified by Dr. Saleem Ahmad (curator at the Herbarium of Pakistan, Museum of Natural History, Islamabad). Voucher specimen (# 032564) of a plant was submitted at the Herbarium of Pakistan, Museum of Natural History, and Islamabad.

### Preparation of plant extract

Leaves of *M. royleanus* were dried in an aerated but shaded area. Dried material was ground by an electric grinder to obtain 60 μm powder. The methanol extract was obtained by allowing 3 kg of powder to macerate 3 times in 95% methanol (3 × 2000 ml) for 5 consecutive days. The supernatants were mixed and filtered. The solvent was evaporated by rotary vacuum evaporator (Buchi, R114, Switzerland). The residue was taken to dryness to obtain a viscous mass as the crude methanol extract (MEM).

### Sample preparation

Stock solutions of quercitin, gallic acid, luteolin, viteixin, apigenin, kaempherol, hyperoside and myricetin were prepared in methanol at concentration of 1 mg/ml and then serially diluted with methanol to get 10, 20, 50, 100 and 200 μg/ml for making the standard calibration curve. MEM stock was prepared as 10 mg/ml in methanol.

### High performance liquid chromatography

HPLC was performed with an Agilent liquid chromatography system, consisting of UV-VIS Spectra-Focus detector (220 nm) and injector-auto sampler. Sample (10 mg/ml) were filtered through a 0.45 μm PVDF-filter and injected in to the HPLC column. The injection volume was 10 μl and the column was 20RBAX ECLIPSE, XDB-C18, (5 μm; 4.6 × 150 mm, Agilent USA). The method involved the use of a binary gradient with mobile phases containing: solvent A (0.05% trifluoroacetic acid) and solvent B (0.038% trifluoroacetic acid in 83% acetonitrile (v/v) with the following gradient: 0–5 min, 15% B in A, 5–10 min, 50% B in A, 10–15 min, 70% B in A. The flow rate was kept constant at 1 ml/min. Identifications were based on retention times in comparison with authentic standards. The crude extract was partitioned three times with 25% hydrochloric acid and methanol. The percolate was concentrated in a rotary evaporator and dissolved in HPLC grade methanol. Calibration curves for standard analytes at 10, 20, 50, 100 and 200 μg/ml concentrations were found to be linear (Supplementary File 1). Quantification of each constituent was completed by means of integration of peaks using the external standard scheme.

### Animals

Adult BALB/c male mice (25–30 g) were obtained from National Institute of Health (NIH), Islamabad and housed at the Primate Facility of Quaid-i-Azam University Islamabad at a temperature of 25 ± 3 °C with a 12 h dark/light cycle in pathogen free environment. They were allowed to standard laboratory feed and water. The study was performed with acceptance (#0173) from the Institutional Animals Ethics Committee, Quaid-i-Azam University Islamabad.

### Assessment of acute toxicity in mice

The acute toxicity assessment was done as per the guidelines 425 of the Organization for Economic Cooperation and Development (OECD) for analysis of chemicals for acute oral toxicity [16]. Mice were separated into six groups with four mice per group. MEM was administered orally as a single dose to mice at different dose levels of 250, 500, 1000, 1500, 2000 and 4000 mg/kg body weight. The animals were examined individually for the signs of toxicity and behaviour for 24 h; at 15, 30, 60 min and 4 h. Animals were observed continuously for 24 h for behavioral, neurological and autonomic profiles and after a period of 14 days for any lethality or death [17].

### RIPE induced hepatotoxicity in mice

Mice were separated randomly in 6 groups with 6 mice in each.

Control (Group I) was treated with vehicle (2% DMSO dissolve in saline, i.p).

Group II received fixed-dose combination of anti-TB drugs [rifampicin (13.5 mg/kg), isoniazid (6.75 mg/kg), pyrazinamide (36.0 mg/kg) and Ethambutol (24.8 mg/kg); RIPE] suspension in sterile saline solution (0.9%) daily for 15 days.

Animals of Group III (200 mg/kg) and Group IV (400 mg/kg) received intraperitoneal treatment of *MEM dissolved in 2% DMSO.*

Group V received vitamin B_6_ at 180 mg/kg dissolved in 10% DMSO, 45 min prior to RIPE challenge.

Group VI was treated with vitamin B6 (180 mg/kg).

Group VII with MEM (400 mg/kg) alone once daily for 15 days**.**

After the last dose of treatment schedule, animals were fasted for 12 h and euthanized by decapitation. The liver was taken out immediately, rinsed in cold saline at 4 °C and blotted dry. One part of a liver was stored at − 70 °C in liquid nitrogen for the determination of biochemical as well as DNA fragmentation assays. The other portion was used for histopathological studies. Blood was withdrawn through cardiac puncture and sera were separated without additive by centrifugation (640 *g*) at 4 °C and stored at − 20 °C for biochemical evaluation.

### Biochemical studies of serum and lipid profile

In the serum the level of AST, ALT, ALP, γ-GT, LDH (aspartate transaminase, alanine transaminase, alkaline phosphatase, gamma glutamyltransferase. Lactate dehydrogenase), total protein, albumin and total bilirubin with AMP diagnostic kits according to the experimental design of the manufacturer. Furthermore, the amount of total cholesterol (TC), high-density lipoproteins (HDL), low-density lipoproteins (LDL) and triglycerides (TG) were approximated by using standard AMP diagnostic kits (Stattogger Strasse 31b 8045 Graz, Austria).

#### Hepatic antioxidant studies

##### Preparation of homogenate and estimation of protein

The liver sections were homogenized in 50 mM phosphate buffer (pH 7.8), the protein content of tissue homogenate was assessed by using bovine serum albumin as standard [18]. The reduced glutathione (GSH) concentration was also estimated in the homogenate. Later on, centrifugation of the homogenate at 9000 *g* (15 min and 4 °C) was done and obtained supernatant was utilized for the estimation of an activity level of various enzymes.

##### Peroxidase (POD) determination

The activity level of the POD enzyme assay was performed by following previous protocol [19]. The variation in absorbance of the sample was determined at 470 nm after each 20 s. An alteration in absorbance of 0.01unit/min was determined as one-unit POD level of activity.

##### Catalase (CAT) assay

The activity level of CAT was estimated by applying the previously established protocol [20]. Alteration in absorbance of the sample at a wavelength of 240 nm followed after every 30 s. Catalase activity one unit was defined as an alteration in absorbance (0.01 unit/min).

##### Superoxide dismutase (SOD) assay

Activity level of SOD in hepatic tissues was determined by well establish protocol [21]. Superoxidase dismutase activity was estimated by the use of sodium pyruvate phosphate and phenazine methosulphate. 150 μL of the supernatant was mixed with 600 μL of 0.052 mM of sodium pyrophosphate buffer (pH 7.0) having 50 μL of 186 mM phenazine methosulphate as substrate. In order to initiate the reaction 100 μL of 780 μM of NADH was added and after 1 min the reaction was pause by the addition of 500 μL of acetic acid. Change of color was determined at 560 nm and SOD activity was evaluated as unit/mg protein.

##### Glutathione-S-transferase (GST) assay

The activity level of GST was determined by the previously reported protocol [22]. The reaction formulation was prepared by the addition of solution comprised of 150 μL of the tissue homogenate to 720 μL of 0.1 mM sodium phosphate buffer in addition to 150 μL of 1 mM GSH and 14.5 μL of chloro-2,4-dinitrobenzene (CDNB). The optical density of the CDNB conjugate formed was determined at 340 nm with a spectrophotometer. The activity level of GST was estimated by the molar coefficient of 9.6 × 10^− 3^/M/cm in terms of CDNB conjugate produced per minute per mg protein.

##### γ-Glutamyltranspeptidase (γ-GT) assay

For the determination of γ-GT activity level of hepatic samples, the glutamylnitroanilide was used as substrate according to the previous protocol [23]. For this purpose, 40 μL of hepatic supernatant was mixed to 200 μL of 5 mM of glutamylnitroanilide, 200 μL of 20 mM glycine and 200 μL of 12 mM of MgCl_2_ mixed in 185 mM of Tris HCl buffer. The reaction formulation was maintained at room temperature for 15 min and the reaction was stopped by the mixing of 200 μL of 30% trichloroacetic acid. The optical absorbance of the homogenate after centrifugation at 2500 *g* for 15 min was observed at 400 nm with a spectrophotometer. The activity of **γ**-GT was evaluated as nM p-nitroaniline produced per minute per mg protein by using the molar coefficient of 1.74 × 10^− 3^/M/cm.

##### Glutathione peroxidase (GPx) assay

The GPx activity level of liver sections was estimated by using previously reported protocol [24]. The reaction formulation was prepared by the adding 1.59 mL of 0.2 M of potassium phosphate buffer (pH 7.4), 0.1 mL of 1 mM of sodium azide, 0.5 mL of glutathione reductase (1 1 U/mL), 0.1 mL of 0.2 mM of NADPH, 0.01 mL of 0.25 mM of H_2_O_2_, and 0.1 mL of the liver sample supernatant by means of reagents like NaN_3_ (1 mM), glutathione reductase (1 IU/mL), NADPH (0.2 mM) and hydrogen peroxide (0.25 mM, 0.01 mL) as reaction substrate. Activity level of GPx was determined as nM NADPH oxidized per minute per mg protein by applying molar coefficient of 6.22 × 10^− 3^/M/cm.

##### Glutathione reductase (GSR) assay

The activity level of GSR was estimated by previously developed protocol [25]. The reaction formulation was done by the addition of 0.2 mL of liver supernatant in a mixture consisted of 1.56 mL of 0.2 M of potassium phosphate buffer (pH 7.6), 0.1 mL of 0.1 mM of EDTA, 0.05 mL of 2 mM of oxidized glutathione and 0.1 mM of NADPH (0.1 mL). The absorbance of the reaction mixture was observed by spectrophotometer at 340 nm at 30 °C. GSR activity was calculated in terms of nM NADPH oxidized per minute per mg protein by using the molar coefficient of 6.22 × 10^− 3^/M/cm.

##### Reduced glutathione (GSH) assay

The concentration of reduced glutathione in liver samples was observed by following the previously reported protocol [26]. 1.0 mL of the liver supernatant was precipitated with 1.0 mL of 4% of sulfosalicylic acid and spun at 1200 *g* for 20 min after placing the sample at 4 °C for 1 h. From the homogenate 0.1 mL was added to a reaction formulation which consisted of 2.8 mL 0.1 M of potassium phosphate buffer (pH 7.4) and 2 mL of 100 mM of dithiobis nitro benzoic acid (DTNB). The absorbance of the yellow product was recorded at 412 nm. The quantity of GSH was estimated as μM GSH/g of liver sample.

#### Oxidative stress markers

##### Lipid peroxidation (TBARS) assay

Determination of lipid peroxidation in liver samples was done as previous procedure [27]. The reaction formulation for the estimation of lipid peroxidation was compiled by adding 0.2 mL of supernatant in the reaction having 0.2 mL of 150 mM of ascorbic acid, 0.02 mL of 100 mM of ferric chloride and 0.58 mL of 0.1 M of potassium phosphate buffer. Afterward, incubated in shaking water bath at the temperature of 37 °C for duration of 1 h. Centrifugation of the reaction mixture was carried out for 15 min and the absorbance of the supernatant was observed at 535 nm against a reagent blank. The quantity of thiobarbituric acid reactant substances (TBARS) was estimated as nM TBARS per min per mg of tissue by applying a molar coefficient of 1.56 × 10^− 5^/M/cm.

##### Hydrogen peroxide (H_2_O_2_) assay

The previously reported protocol was followed for determination of hydrogen peroxide in the liver samples [28]. The cocktail of the reaction comprised of 0.1 mL of supernatant, 0.5 mL of 0.05 M of phosphate buffer (pH 7), 0.1 mL of 0.28 nM phenol red, 0.25 mL of 5.5 nM of dextrose and 8.5 units of horseradish peroxidase. The reaction formulation was placed at room temperature for 60 min followed by the addition of 0.1 mL of 10 N of NaOH to terminate the reaction. The absorbance of the supernatant was observed at 610 nm after centrifugation of the reaction formulation at 800 *g* for 10 min. Amount of hydrogen peroxide produced was determined as nM H_2_O_2_ per min per mg tissue by a applying reference curve of hydrogen peroxide oxidized phenol red.

##### Hepatic histopathological studies

Hepatic sections were dehydrated in ascending order of ethyl alcohol, shifted to cedar wood oil, fixed in paraffin, thin sections of 3–5 μm were prepared and hematoxylin-eosin was used for staining purpose. Histopathological studies were conducted with a microscope (DIALUX 20 EB) and photographed were captured at 400 × magnification.

##### DNA damaging studies

Liver tissues preserved in liquid nitrogen were used for the determination of DNA damages by applying the previous standard experimental design [26]. Hepatic tissues (100 mg) were homogenized in 10 volumes of TE (pH 8.0) solution (5 mM Tris-HCl; 20 mM EDTA) and 0.2% of Triton X-100. It was spun at 27000 *g* for 20 min to attain the pellet (B) and the supernatant (T). The quantity of DNA in the pellet and the supernatant was estimated at 620 nm with spectrophotometer by using the diphenyl-amine solution.

$$ \% Fragmented\  DNA=\frac{\mathrm{Supernatant}}{\mathrm{Supernatant}+\mathrm{Pellet}} \times 100 $$

##### DNA ladder protocol+

For the determination of DNA damages 1.5% agarose gel comprising of 1.0 μg/mL ethidium bromide (EtBr) was used. In each well 5 μg of genomic DNA while 0.5 μg the marker DNA was utilized to determine the DNA damages. Subsequently, the image of a gel was observed in the gel doc system [29].

### Statistical analysis

Data are presented as Mean ± SEM (*n* = 6). Statistical differences between different treatments were calculated by one-way analysis of variance (ANOVA) followed by Tukey’s test on Graph pad prism 5 software. Significance level was set at *p* < 0.05.

## Results

### HPLC analysis of MEM

**Fig. 1:**
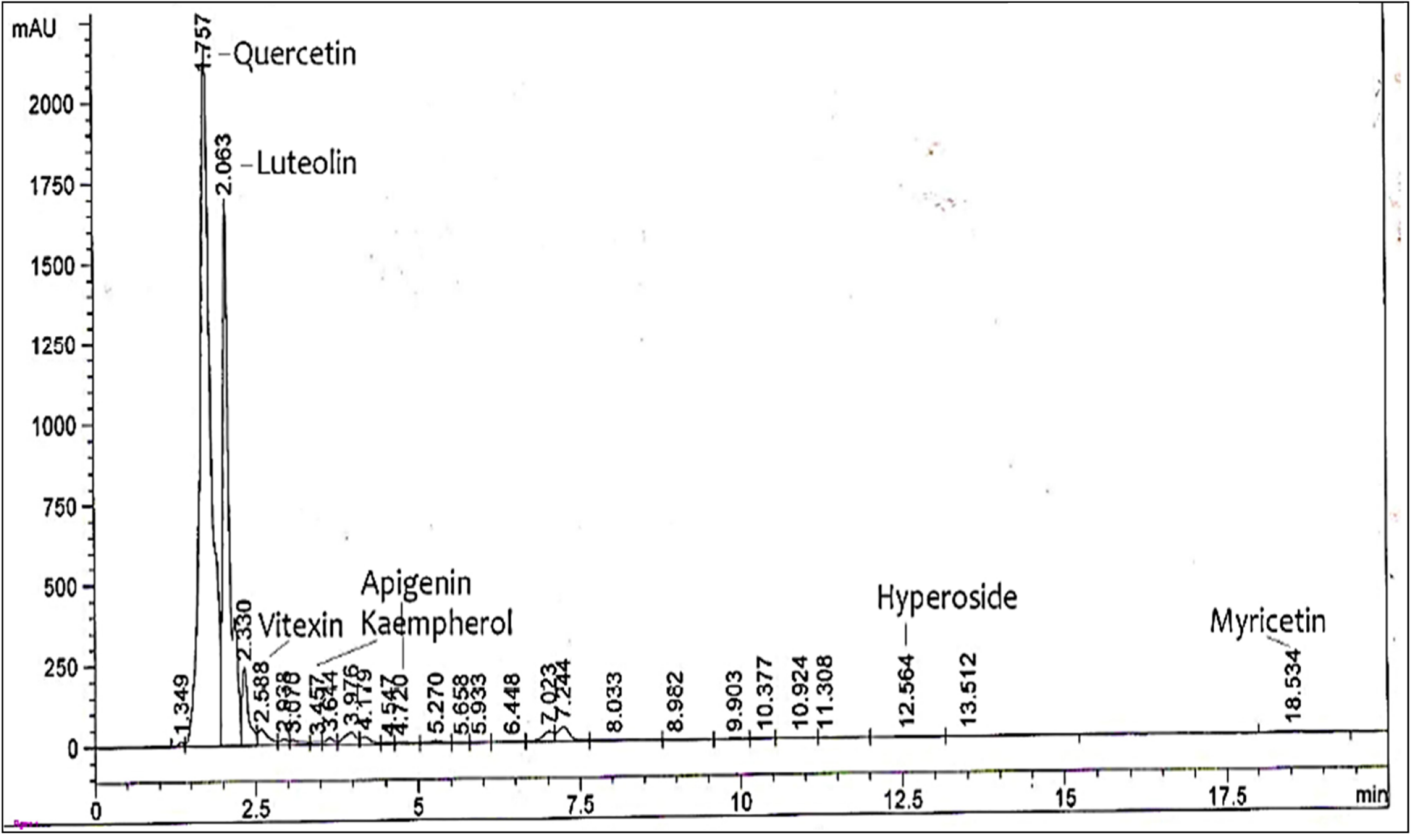
HPLC chromatogram of MEM

Figure 1 indicated the HPLC chromatogram of MEM. Based on a comparison of the retention times with those of the standards, major peaks 1 and 2 were identified as quercetin and luteolin. The other compounds are detected in minor quantities as indicated by low pea areas, hence quercetin and luteolin are shown to be major flavonoids identified in MEM. The concentration of quercetin and luteolin in MEM is estimated to be (0.217 mg/g dry weight) (0.141 mg/g dry weight) respectively.

### Effect of methanol extract of *M. royleanus* leaves on liver and body weight in mice

Anti-TB drug (RIPE) treated group significantly decrease the liver weight compared to control group. MEM treatment markedly ameliorated the effect of anti-TB drug on liver weight. Impact of MEM was comparable to the effect of vitamin B6 on hepatic weight. Similarly, final body weight was significantly decreased with treatment of anti-TB drug. MEM treatment significantly ameliorated RIPE induced decline in body weight. On the contrary, a significant weight gain was observed with the vitamin B_6_ and MEM co-treated groups (Table 1).

**Table 1:** Protective effect methanol extract of *M. royleanus* leaves on liver and body weight of mice

Treatments	Liver weight (g)	Initial body weight (g)	Final body weight (g)
Control	6.22 ± 1.36	25.53 ± 0.58	34.7 ± 0.26
RIPE	4.80 ± 0.74^*^	25.33 ± 0.46	27.1 ± 0.30^***^
RIPE + MEM (200 mg/kg)	5.71 ± 0.97^+^	24.83 ± 0.51	31.0 ± 0.39^***, +++, ##^
RIPE + MEM (400 mg/kg)	5.98 ± 0.13^+^	25.21 ± 0.39	33.1 ± 0.41^+++^
RIPE + Vit B_6_	5.51 ± 1.81^+^	25.11 ± 0.52	33.4 ± 0.29^+++^
Vit B6 (180mg/kg) alone	6.28 ± 1.74^+^	25.30 ± 0.47	35.1 ± 0.30^+++^
MEM (400 mg/kg) alone	6.06 ± 1.13^+^	24.71 ± 0.53	34.8 ± 0.37^+++^

Values are represented as mean ± SEM (*n* = 6). Data analyzed by one-way analysis of variance followed by multiple comparison test. Asterisks *, **, *** represents significance from control group at *p* < 0.05, *p* < 0.001, and *p* < 0.0001. +, +++ represents significance from RIPE group at *p* < 0.05 and *p* < 0.0001 while ### represents significance difference of RIPE + MEM (200 mg/kg) vs RIPE + MEM (400 mg/kg) group at *p* < 0.0001

### Lipid profile

Liver toxins reacts with polyunsaturated fatty acids to encourage lipid peroxidation by disturbing the lipid profile [30]. RIPE treatment evidently (*P* < 0.001) augmented the quantity of total cholesterol, LDL and triglycerides, while decreasing (*P* < 0.001) HDL levels as compared to control group (Table 2). Co-treatment of MEM with RIPE, dose dependently improved the abnormal lipid profile. MEM high dose treated group showed similar shielding action against RIPE induced lipid profile changes as shown by Vitamin B6 treated group. Animals treated with AHE alone at 400 mg/kg.bw dose showed an insignificant difference in results compared to control group.

**Table 2:** Protective effects of MEM on Serum Lipid Profile

Treatments	Total Cholesterol (mg/dl)	Triglycerides (U/L)	HDL (mg/dl)	LDL (mg/dl)
Control	62.37 ± 2.61	54.43 ± 0.74	40.77 ± 2.27	29.1 ± 3.19
RIPE	98.10 ± 1.25^****^	134.09 ± 3.35^****^	22.78 ± 2.41 ^****^	59.18 ± 1.79^****^
MEM (200 mg/kg) + RIPE	80.77 ± 2.98^****++++^	72.91 ± 5.93^****++++^	31.80 ± 1.09**^++++##^	44.31 ± 1.27^****++++^
MEM (400 mg/kg) + RIPE	77.40 ± 3.74^****++++^	67.81 ± 1.17^****++++^	38.78 ± 2.43^++++^	33.37 ± 3.69^++++^
Vit B6 + RIPE	70.91 ± 2.69^****++++^	63.30 ± 3.07**^++++^	39.01 ± 1.39^++++^	34.01 ± 2.07^++++^
Vit B6	63.98 ± 1.37^++++^	55.79 ± 0.73^++++^	41.01 ± 1.59^++++^	28.02 ± 0.31^++++^
MEM (400 mg/kg)	65.80 ± 1.11^++++^	59.37 ± 5.14^++++^	42.11 ± 0.94^++++^	29.11 ± 3.11^++++^

Values are Mean ± SD (06 number). RIPE, Rifampicin (13.5 mg/kg), Isoniazid (6.75 mg/kg), Pyrazinamide (36.0 mg/kg) and Ethambutol (24.8 mg/kg). MEM, *M. royleanus* leaves methanol extract. *, **, *** indicate significance from the control group at p < 0.05, *p* < 0.01 and p < 0.0001 probability level, +, ++, +++ indicate significance from the RIPE group at *p* < 0.05, *p* < 0.01 and *p* < 0.0001, while ### indicate significance of MEM (400 mg/kg) + RIPE group vs MEM (200 mg/kg) + RIPE group at p < 0.0001 probability level (One-way ANOVA followed by Tukey’s multiple comparison tests)

### Effect of MEM on liver function tests (LFTs)

RIPE administration increased the concentration of AST, ALT, ALP, γ-GT, and LDH in serum compared to the control group. The hepatotoxicity induced with RIPE was ameliorated by the co-administration of MEM to RIPE administered mice. The protective effects of MEM on AST, ALT, ALP, GT and LDH were produced in a concentration dependent manner. The level of AST, ALT, ALP, γ-GT, and LDH in the serum of MEM and vitamin B6 alone administered groups remained unaffected compared to the control group (Table 3).

**Table 3:** Protective effects of MEM on liver marker enzymes in serum of mice

Group	AST (U/l)	ALT (U/l)	ALP (U/l)	γ-GT (U/l)	LDH (U/l)
Control	94.17 ± 3.41	70.46 ± 2.35	142.87 ± 4.65	1.84 ± 0.43	45.34 ± 1.61
RIPE	204.9 ± 6.11^****^	211.2 ± 5.13^****^	306.47 ± 6.68^****^	4.01 ± 0.75^****^	141.8 ± 4.17^****^
MEM (200 mg/kg) + RIPE	170.29 ± 6.38^****++++####^	169.9 ± 4.28^****++++####^	252.14 ± 5.31^****++++####^	2.98 ± 0.24^*+^	110.5 ± 3.68^****++++####^
MEM (400 mg/kg) + RIPE	121.15 ± 4.28_****++++_	123.0 ± 2.24^****++++^	172.13 ± 3.20^****++++^	2.16 ± 0.12^++++^	73.26 ± 2.34^****++++^
Vit B6 + RIPE	109.62 ± 4.4^****++++^	98.13 ± 3.13^****++++^	161.15 ± 3.98^****++++^	2.34 ± 0.57^++++^	72.1 ± 2.75^****++++^
Vit B6 alone	98.26 ± 4.89^++++^	68.14 ± 2.81^++++^	143.66 ± 3.63^++++^	1.98 ± 0.46^++++^	44.32 ± 2.23^++++^
MEM (400 mg/kg) alone	93.73 ± 4.36^++++^	69.58 ± 2.63^++++^	144.35 ± 5.87^++++^	1.93 ± 0.79^++++^	47.16 ± 1.49^++++^

Values are Mean ± SD (06 number). RIPE, Rifampicin (13.5 mg/kg), Isoniazid (6.75 mg/kg), Pyrazinamide (36.0 mg/kg) and Ethambutol (24.8 mg/kg). MEM, *M. royleanus* leaves methanol extract. Vit B6, Vitamin B6. *, **, *** indicate significance from the control group at p < 0.05, p < 0.01 and p < 0.0001 probability level, +, ++, +++ indicate significance from the RIPE group at *p* < 0.05, *p* < 0.01 and *p* < 0.0001, while ### indicate significance of MEM (400 mg/kg) + RIPE group vs MEM (200 mg/kg) + RIPE group at p < 0.0001 probability level (One-way ANOVA followed by Tukey’s multiple comparison tests)

### Effect of MEM on antioxidant enzymes

Effect of MEM on RIPE induced deterioration of tissue antioxidant enzymes is reveled in Table 4. The activity level of phase I antioxidants such as CAT, POD and SOD is decreased (*p* < 0.05) as compared to the control group. The protective potential MEM against the toxicity induced with RIPE on the phase I antioxidant enzymes was evident by significant (*p* < 0.05) increase in CAT, POD and SOD compared to RIPE treated group. Co-administration of vitamin B6 along with RIPE to mice ameliorated the toxicity of RIPE and increased the level of CAT, POD, and SOD in liver samples as compared to the RIPE treated group. The protective effects of MEM at 400 mg/kg dose were comparable (*p* > 0.05) to the vitamin B6 treated group for the CAT and SOD activity while for POD activity level the vitamin B6 + RIPE group exhibited more protective potential (*p* < 0.05) to that of the MEM + RIPE co-treated group. Administration of vitamin B6 (180 mg/kg) to mice elevated (p < 0.05) the activity level of POD and SOD while no effect (p > 0.05) on the activity level of CAT as compared to the control group. MEM (400 mg/kg) administration to mice showed significant (p < 0.05) elevation in the activity level of CAT and SOD whereas the activity level of POD was not influenced (p > 0.05) as compared to the control group.

**Table 4:** Protective effects of MEM on liver antioxidant status

Treatments	CAT (U/min)	POD (U/min)	SOD (U/mg protein)	GSH (nM/g tissue)	GST (μM/mg protein)	GPx (nM/ min/mg protein)	GSR (nM /min/mg protein)	γ-GT (nM /min/mg protein)
Control	1.93 ± 0.16	1.99 ± 0.11	9.23 ± 0.21	40.82 ± 2.07	6.84 ± 0.41	123.20 ± 4.22	211.31 ± 5.22	82.36 ± 3.26
RIPE	0.54 ± 0.11^****^	1.11 ± 0.14^****^	4.15 ± 0.23 ^****^	32.18 ± 1.88^****^	3.58 ± 0.45^****^	59.28 ± 2.22^****^	153.17 ± 4.13^****^	61.42 ± 3.45^****^
MEM (200 mg/kg) + RIPE	1.83 ± 0.10^++++^	1.52 ± 0.13^****+++^	6.54 ± 0.17 ^****++++####^	35.51 ± 2.13^**^	5.35 ± 0.47^****++++^	89.70 ± 3.15^****++++####^	177.12 ± 4.03^****++++####^	68.74 ± 3.87^**** + #^
MEM (400 mg/kg) + RIPE	1.89 ± 0.12^++++^	1.72 ± 0.14^*++++^	8.35 ± 0.19 ^****++++^	37.71 ± 2.26^+++^	5.56 ± 0.34^***++++^	102.07 ± 3.03^****++++^	199.29 ± 6.20^**++++^	76.38 ± 4.21^++++^
Vit B6 + RIPE	2.08 ± 0.12^++++^	2.04 ± 0.13^++++^	8.12 ± 0.24 ^****++++^	36.51 ± 2.05^*+^	6.19 ± 0.67^++++^	99.24 ± 3.40^****++++^	187.30 ± 5.01^****++++^	73.54 ± 3.17^**++++^
Vit B6	1.88 ± 0.14^++++^	2.18 ± 0.15^++++^	11.32 ± 0.18^****++++^	41.46 ± 2.20^++++^	6.36 ± 0.46^++++^	118.50 ± 3.21^++++^	209.40 ± 6.05^a++++^	84.26 ± 4.12^a++++^
MEM (400 mg/kg)	2.42 ± 0.17^**++++^	1.83 ± 0.17^++++^	10.36 ± 0.23 ^**++++^	42.39 ± 1.67^++++^	5.62 ± 0.48^**++++^	119.25 ± 3.59^++++^	201.33 ± 5.02^*++++^	85.65 ± 4.65^a++++^

Values are Mean ± SD (06 number). RIPE, Rifampicin (13.5 mg/kg), Isoniazid (6.75 mg/kg), Pyrazinamide (36.0 mg/kg) and Ethambutol (24.8 mg/kg). MEM, *M. royleanus* leaves methanol extract. Vit B6, Vitamin B6. *, **, *** indicate significance from the control group at p < 0.05, p < 0.01 and p < 0.0001 probability level, +, ++, +++ indicate significance from the RIPE group at *p* < 0.05, *p* < 0.01 and *p* < 0.0001, while ### indicate significance of MEM (400 mg/kg) + RIPE group vs MEM (200 mg/kg) + RIPE group at p < 0.0001 probability level (One-way ANOVA followed by Tukey’s multiple comparison tests)

Activity level of phase II antioxidant enzymes such as GSH, GST, GPx, GSR and γ-GT decreased (p < 0.05) with the RIPE administration as compared to the control group (Table 3). Toxico-suppressive effects of MEM against the hepatotoxicity with RIPE were recorded and the activity level of GSH GST, GPx, GSR and γ-GT in hepatic samples of mice increased as compared the RIPE treated group. Co-treatment of vitamin B6 with anti-tuberculosis drug administered mice elevated the activity level of antioxidant enzymes in hepatic samples in comparison to the RIPE administered mice group. Activity level of GPx and γ-GT with the treatment of MEM (400 mg/kg) alone was statistically similar (*p* > 0.05) to the control group while activity level of GST and GSR decreased (*p* < 0.05) as compared to control group.

### Effect of MEM on hepatic protein content and oxidative stress markers

The protective potential of MEM against the RIPE induce oxidative stress and protein content is presented in Table 5. RIPE administration decreased the soluble concentration of protein in hepatic samples of mice compared to the control animals. The mice co-treated with vitamin B6 and MEM along with RIPE exhibited the increase in soluble protein concentration in tissues compared to the mice treated with anti-tuberculosis drug alone. The protective ability of the MEM on soluble protein concentration was in dose dependent manner. Administration of vitamin B6 and MEM alone to mice non-significantly elevated the level of protein compared to the control mice Treatment with RIPE significantly increase hepatic oxidative stress as depicted by increased concentration of TBARS and H_2_O_2_ in hepatic tissues compared to the control group. MEM treatment in combination with RIPE lessened the toxic effects of anti-tuberculosis drug and decreased the concentration of TBARS and H_2_O_2_ in hepatic samples in a dose dependent manner. The ameliorative potential of MEM is equivalent to standard vitamin b_6_ treated group.

**Table 5:** Protective effects of MEM on liver protein and oxidative stress markers

Treatments	Protein (μg/mg tissue)	TBARS (nM of MDA/mg protein)	H_**2**_O_**2**_ (nM/min /mg tissue)
Control	1.32 ± 0.16	6.09 ± 1.30	5.38 ± 0.42
RIPE	0.73 ± 0.12^****^	18.65 ± 3.72^****^	8.88 ± 1.04^****^
MEM (200 mg/kg) + RIPE	1.10 ± 0.15^++^	13.65 ± 1.82^****++#^	6.98 ± 0.80^**+++^
MEM (400 mg/kg) + RIPE	1.21 ± 0.13^++++^	9.78 ± 1.31^*++++^	5.78 ± 0.58^++++^
Vit B6+ RIPE	1.16 ± 0.18^+++^	9.43 ± 1.55^c++++^	6.54 ± 0.66^++++^
Vit B6	1.41 ± 0.18^++++^	5.80 ± 0.80^e++++^	4.95 ± 0.60^++++^
MEM (400 mg/kg) alone	1.38 ± 0.15^++++^	7.07 ± 1.79^d++++^	5.44 ± 0.52^++++^

Values are Mean ± SD (06 number). RIPE, Rifampicin (13.5 mg/kg), Isoniazid (6.75 mg/kg), Pyrazinamide (36.0 mg/kg) and Ethambutol (24.8 mg/kg). MEM, *M. royleanus* leaves methanol extract. Vit B6, Vitamin B6. *, **, *** indicate significance from the control group at p < 0.05, p < 0.01 and p < 0.0001 probability level, +, ++, +++ indicate significance from the RIPE group at p < 0.05, p < 0.01 and p < 0.0001, while ### indicate significance of MEM (400 mg/kg) + RIPE group vs MEM (200 mg/kg) + RIPE group at p < 0.0001 probability level (One-way ANOVA followed by Tukey’s multiple comparison tests)

### Effect of MEM on histopathology of liver

**Fig. 2:**
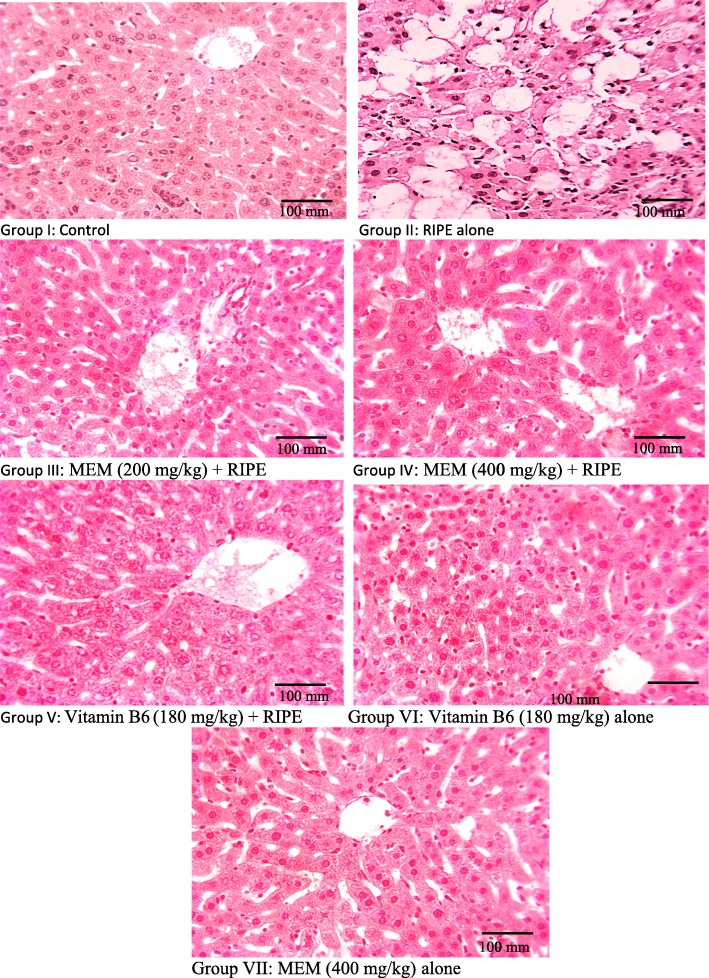
H & E stain; × 400. (I): Representative section of liver from the control group showing the normal architecture, (II): RIPE treated group (III) MEM (200 mg/kg) + RIPE treated group, (IV): MEM (400 mg/kg) + RIPE treated group, (V): Vitamin B6 (180 mg/kg) + RIPE treated group, (VI): Vitamin B6 (180 mg/kg) treated group and (VII): MEM (400 mg/kg) treated group

The histological architecture of the control group showed the normal lobular structure of liver (Fig. 2). In anti-tuberculosis drug treated mice, the histopathology of the liver was altered and fatty changes were prominent. The lobular structure was disrupted and there was congestion of blood vessels, a severe degree of hemorrhage, necrosis with fatty vacuolations. There were degenerative changes and the chromatin material showed clumped morphology. The cell membrane hepatocytes in some of the areas were not distinguished. Treatment of mice with MEM protected the liver from the toxicity of anti-tuberculosis drug and most of the changes induced with an anti-tuberculosis drug were absent from the histopathology. Low level of toxicity was apparent in the MEM (200 mg/kg) treatment, while at a higher dose (400 mg/kg) the histopathological architecture of liver was near to the control mice. In the case of vitamin B6, the histopathological alterations induced with the anti-tuberculosis drug were recorded at a lower level and hepatocytes presented a minor level of toxicity. Administration of vitamin B6 alone to mice caused a foamy appearance of hepatocytes while in case of MEM (400 mg/kg) dose to mice an almost normal architecture of liver was apparent. Furthermore, MEM alone treatment seems to be safe in all organs of mice. The pathologist report has been attached in supplementary file 2.

### Effect of MEM on DNA damage

**Fig. 3:**
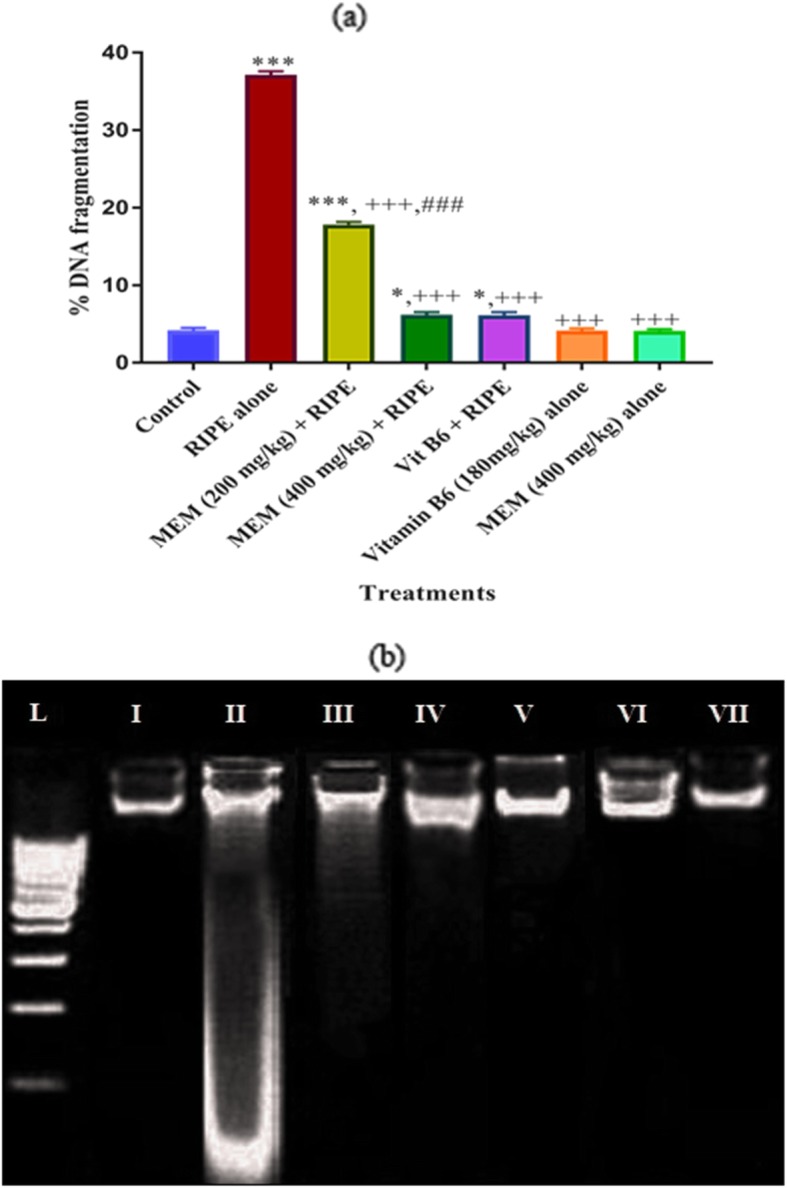
Lane from left to right; L, 100 bp ladder (low molecular weight DNA marker) (I) Control group (II) RIPE treated group (III) MEM (200 mg/kg) + RIPE treated group (IV) MEM (400 mg/kg) + RIPE treated group, (V) Vitamin B6 + RIPE treated group, (VI) Vitamin B6 alone treated group (VII) MEM (400 mg/kg) alone treated group. MEM: *Maytenus royleanus* methanol extract. RIPE: 13.5 mg/kg Rifampicin, 6.75 mg/kg Isoniazid, 36.0 mg/kg Pyrazinamide and 24.8 mg/kg Ethambutol

Analysis of DNA damage in liver tissues by percent DNA fragmentation and ladder assay is presented in Fig. 3 (a, b). RIPE administration induce significantly high percent DNA fragmentation. Co-administration of RIPE treated rats with MEM significantly attenuated DNA fragmentation in a dose dependent fashion. The effect of MEM high dose is similar to Vitamin B6 treated group (Fig. 3a). DNA ladder assay revealed that the DNA remain entangled at the base of the well and showed a sharp single band without degradation and tail pattern in liver tissues of the control as well as in the vitamin B6 and MEM alone treated groups (Fig. 3b). Treatment of mice with RIPE induced DNA damages in liver tissues and showed continuous pattern of DNA fragmentation in ladder assay. DNA isolated from the liver tissue of mice treated with MEM + anti-tuberculosis drug showed significant protection from DNA damage as revealed by sharp DNA band similar to untreated group.

## Discussion

Anti-TB drugs are the most common group of drugs that are known to cause severe hepatotoxicity worldwide and overall, hepatotoxicity attributed to anti-TB drugs has been reported in 5–28% of people treated with anti-TB drugs. Up to 20% of the patients receiving RIPE either in single or combination therapy develop transient asymptomatic elevation in liver enzymes, which settle with continued use of the drug and its hepatotoxicity manifestation can vary from asymptomatic elevations in the liver enzymes to fulminant liver failure. Eras of medical surveillance have recognized a range of drugs as well as host linked aspects which are related to an enhanced threat of anti-tuberculous drug-prompted liver injury [8]. Liver biopsy specimens reveal lobular hepatitis, sub massive to massive necrosis and hydropic degeneration of hepatocytes in severe cases [31].

In current investigation the commonly used anti-tuberculosis (antiTB) drugs; pyrazinamide (PZA), isoniazid (INH), ethambutol (ETB) and rifampicin (RMP) were administered to mice in order to investigate toxic consequences of combination therapies on liver. The outcome of the present study represents the induction of severe hepatocellular injuries as evidenced by disturbed liver profile and upregulation of AST, γ-GT, ALP, ALT, and LDH in the serum of mice. Hepatotoxicity induced with the anti-TB drug was ameliorated by co-administration of MEM and the level of LDL, cholesterol, triglycerides, AST, ALT, ALP, γ-GT, and LDH in the serum of mice decreased in dose-dependent manner suggesting the protective ability of MEM. The protective effect of MEM might be due to the presence of bioactive metabolites (Table 6). As earlier reports signify that quercetin and luteolin improve lipid profile and inhibit inflammatory cytokines [32, 33]. Administration of MEM (400 mg/kg) alone to mice did not induce an alteration in the level of hepatic biomarkers enzymes, signifying the safe effect of MEM. The co-administration of vitamin B6 to the anti-TB drug administered mice decreased the level of AST, ALT, ALP, γ-GT, and LDH in the serum of mice suggesting the protective effects on liver function. The administration of Vitamin B_6_ alone to mice did not induce a noteworthy alteration in the level of ALT, ALP, γ-GT and LDH in serum compared to control animals. However, administration of vitamin B6 causes a non-significant escalation in the level of AST in the serum of mice compared to the control group. The increase in AST might be attributed to the deficiency of vitamin B6 in mice [34]. Induction of hepatic injuries with the anti-TB drug is multifaceted, but the major mechanism seems to be oxidative stress induced generation of free radicles. In this investigation, the anti-TB drug to mice shifts the dynamic equilibrium of metabolism towards the oxidative stress as observed by an increase of hepatic TBARS and H_2_O_2_ while a decrease in hepatic GSH and the antioxidant enzymes. Insufficient hepatic level of CAT, SOD, and GPx was unable to scavenge the excessive generation of lipid hydroperoxides. These reactive intermediate metabolites play a crucial role in developing oxidative stress and consequently cause hepatic damages [35]. Amongst the cell based antioxidants GST, CAT, POD, GR, SOD, and GPx are extensively explored for their significant job in defense mechanism. Superoxidase is an exceptionally active antioxidant enzyme that catalyzes the dismutation reaction of superoxides to H_2_O_2_ and O_2_ whereas CAT is a ubiquitous enzyme but mainly rich in the liver and is engaged in a breakdown of H_2_O_2_ to water. In the GSH reaction system, GSH is oxidized to GSSG by the help of GPx which transformed back to GSH by the reducing power of GSR. GSH also works as a cofactor for GST that is present equally in the cytosol and endoplasmic reticulum, essentially engage in catalyzing the production of GSH electrophile conjugate therefore, detoxifying xenobiotics to generate irreversible compounds. It is detected that lipids peroxidation can induce a genetic increase of fibrogenic cytokines by commencing the generation of collagen and stimulating liver stellate cells [36]. Enhanced Complement system activation is known for tissue injuries and free radicals activates complement system. More complement activation causes more C3b generation and that can lead to tissue injuries [37] . In the current research, the level of TBARS and H_2_O_2_ content moved in the direction of control after treatment with MEM. This refurbishment may be accompanied with improvement of the antioxidant enzymes. The decrease of TBARS and increase of GSH in hepatic samples have been determined with the co-administration of *Bombax ceiba* extract to anti-TB drug administered rats [38]. Our findings are relevant to other observations about hepatic tissue [39]. Methanol extract of *M. emarginata* has antioxidant potential evaluated by SOD, DPPH, ABTS, iron chelating and free radical NO quenching assays [40]. Another study revealed that *M. krukovii* hydro-alcoholic extract of bark possessed an inhibitory potential against the mutation causing activity (promutagens) of 2-aminoanthracenein in both T98 and T100 strains on the other hand showed poor activity towards mutagens such as sodium azide and 2-nitrofluorene. *M. krukovii* demonstrated scavenging ability depending on the concentration of dose. A potential antioxidant activity against the monocation 2, 2′-azinobis (3-ethylbenzothiazoline-6-sulfonic acid) and HOCl- was shown by the ethanol extract of M. ilicifolia root [41].

**Table 6:** Extraction yield, TPC, TFC, and chemical constituents in M.roelyanus leaves extract (MEM)

Analysis (MEM extract)	Observations	(References)
Extraction yield (%)	12.2%	[11]
TPC (mg gallic acid equivalent/g dry sample)	76 ± 2.7	[11]
TFC (mg rutin equivalent/g dry sample)	63.5 ± 1.84	[11]
LC-MS Compound Fingerprinting	Glucosinolates, Anthocyanines, Phenolics, Chlorophylls, Macro and micro constituents	[11]
HPLC-DAD (Identification of compounds with reference to standards)	Caffeic acid Quercetin 3 rhamnoside	[12]

*TPC* Total Phenolic content, *TFC* Total flavonoid content. Information derived from our previous lab investigations

Presence of quercetin and luteolin might be responsible or the protected afforded by MEM. Previous studies indicated that quercetin is responsible for substantial defense in contradiction of INH and RFP-induced toxicity in rats liver demonstrated a decrease in AST and ALT potentials, an upsurge in total antioxidant activity, and normal histopathological picture of the rats liver [42]. The vitamin B6 was used as standard antioxidant compound in our study and the effect of MEM was shown to be equivalent to vitamin B6. The protectective effect of Vitamin B6 has been already validated in previous researches. Single dose of vitamin B6 directed right after surgery helped in improved oxidative parameters and inflammatory markers in liver. It results in significant reduction of oxidative stress in liver of mice. The immediate administration of vitamin B6 in mice contributed towards neutrophil reduction in liver and helped in diminishing the oxidative damage to protein and lipids in liver [43]. Vitamin B6 also modulates the kynurenine pathway, sphingosine-1-phosphate, and nuclear factor-kappa B thus lowering inflammation [44]. Hence, the mechanism of vitamin B6 is via regulating the oxidative stress in peripheral organs i.e. liver and lungs etc. Hence we speculated that similarly to Vitamin B6, MEM might have rescued liver injury through modulating oxidative stress in mice liver. Quercetin and luteolin being one of the major phytochemical constituents of *M. royleanus* might be responsible for the hepato-preventive effects.

The histopathology is unswerving technique for evaluating the ability of test samples at tissue level, additionally its provides correlation between the functions of serum biomarkers, tissue enzyme levels and morphological alterations [30]. Noteworthy alterations in liver function tests (LFTs) predominantly epitomizes the fibrosis in liver. Fibrosis not only intrudes the normal morphology but also interferes the flow of blood to preclude the transport of nutrients to liver tissues. Liver histology of anti-TB administered group showed marked histopathological alterations in liver structure, dissolution of hepatic cords, which give the impression of empty vacuoles aligned by strands of necrotic hepatocytes, nuclear disintegration, vacuolar degeneration, apoptotic cell death, fibrosis and collagen deposition in some parts. Our finding is in parallel with previous reports on the toxic effect of anti-TB drug induced liver toxicity. They observed extreme hepatocyte hypertrophy characterized by a notable increase in cell size accompanied by binucleate hepatocytes with enlarged hepatocyte nuclei [45, 46]. The liver tissue samples of MEM treated groups exhibited diminished necrosis, slight inflammatory cells without damage to cell membrane signifying its protective potential. The observed hepatoprotective effect might be correlated to the presence of bioactive compounds in MEM most specifically; quercetin and luteolin. Previous studies also indicated the hepatoprotective action of quercetin and luteolin against Thioacetamide induced biochemical and histological changes in rat liver. The mechanism of protection is through modulating oxidative stress and augmentation of antioxidant enzymes [47, 48]. A recent study indicated that luteolin protect liver injury by inhibition of inflammatory biomarkers [49].

Binding of free radicals can contribute towards the macromolecular injuries like DNA to induce mutation, lipids to induce membrane damages and proteins to change their function. In the current research the hepatic DNA assaulted by anti-TB drug generated free radicals evidenced by the higher level of hepatic damages and in DNA ladder assay. Alternatively, co-treatment of MEM substantially decreased the % DNA fragmentation that was demonstrated by DNA ladder assay banding pattern. Analogous findings were presented in a study of [50] while investigating the scavenging potential of *Sonchus arvensis* in response to carbon tetrachloride intoxicated rat liver. The observed hepatoprotective effect might attribute to the occurrence of phytochemicals as well as various biologically active secondary metabolites i.e. quercetin and luteolin that are the major compounds identified by HPLC [10, 12].

## Conclusion

The outcomes of the current study demonstrated that anti-TB drug induced a harmful effect on the liver by affecting DNA, lipids, and protein, together with generating oxidative stress. MEM demonstrated hepato-protective ability by preventing oxidative DNA damage, by enhancing the potentials of antioxidant enzymatic as well as non-enzymatic levels besides regulating the histological alterations. Serological studies for liver function tests also proved the protective consequence of MEM against anti-TB drug caused deteriorations. These protective effects might be credited by the occurrence of various antioxidant phytochemicals. Future investigations exploring the mechanisms underlying the pathogenesis of anti-TB drug should be performed using human tissue and samples whenever possible, so that the novel findings can be translated readily into clinical applications.

## Supplementary information

**Additional file 1:** Pathology report.

**Additional file 2: Figure S1.** Standard calibration curve of standard compounds.

## Data Availability

All data generated or analyzed during this study are included in this published article. The raw used and/or analyzed during the current study can be available from the corresponding author on reasonable request.

## Abbreviations

MEM: *Maytenus royleanus*
RIPE: Rifampicin (13.5 mg/kg), Isoniazid (6.75 mg/kg), Pyrazinamide (36.0 mg/kg) and Ethambutol (24.8 mg/kg)
MDA: Malonyldialdehyde
CAT: Catalase
TBARS: Thiobarbituric acid reactive substances
SOD: Superoxide dismutase
GPx: Glutathione peroxidase
H_2_O_2_: Hydrogen peroxide
NO: Nitric oxide
CYP2E1: Cytochrome P450 2E1
