# Supplementary figures and images for: Phytochemical analysis and Evaluation of hepatoprotective effect of Maytenus royleanus leaves extract against anti-tuberculosis drug induced liver injury in mice
Source: Lipids Health Dis. 2020 Mar 16;19:46. doi: 10.1186/s12944-020-01231-9 (PMC7077109; doi:10.1186/s12944-020-01231-9)

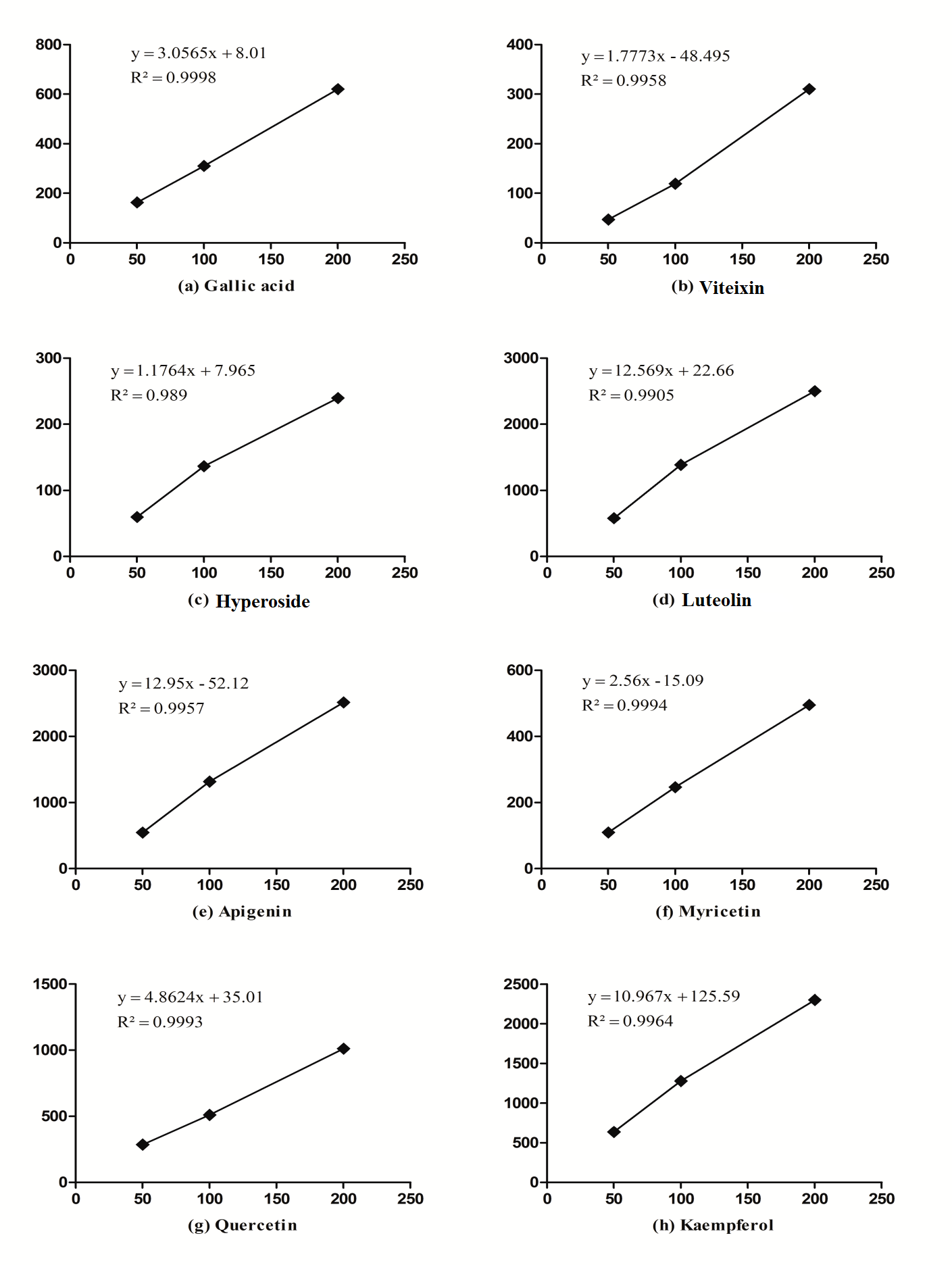


Supplementary Figure 1: Standard calibration curve of standard compounds.

Supplement: Supplementary file 1 — Additional file 1: Pathology report. [file 12944_2020_1231_MOESM1_ESM.docx]
