# Supplementary material for: Phytochemical analysis and Evaluation of hepatoprotective effect of Maytenus royleanus leaves extract against anti-tuberculosis drug induced liver injury in mice
Source: Lipids Health Dis. 2020 Mar 16;19:46. doi: 10.1186/s12944-020-01231-9 (PMC7077109; doi:10.1186/s12944-020-01231-9)
Supplement: Supplementary file 2 — Additional file 2: Figure S1. Standard calibration curve of standard compounds. [file 12944_2020_1231_MOESM2_ESM.docx]

Deeba Syed [dsyed@dermatology.wisc.edu](mailto:dsyed@dermatology.wisc.edu)

Maria Shabbir [mshabbir@dermatology.wisc.edu](mailto:mshabbir@dermatology.wisc.edu)

Slide review, RS 9/15/13

Preliminary observations, Ruth Sullivan, VMD PhD, Diplomate ACVP

**1209**:

Liver- The liver capsule is segmentally mildly to markedly broadened with dense fibrous connective tissue with abundant extracellular matrix that is infiltrated with modest numbers of mononuclear leukocytes and neutrophils. Leukocytes are most prominent at the interface between the fibrotic capsule and the hepatic parenchyma. Here, there are occasional presumptive macrophages with light brown cytoplasmic pigment. A few portal zones are infiltrated with modest numbers of mononuclear leukocytes and occasional presumptive macrophages with light brown cytoplasmic material. There are scattered mitotic hepatocytes. Occasional megakaryocytes and clusters of mononuclear cells resembling hematopoietic elements suggest scattered extramedullary hematopoiesis (EMH). There are occasional scattered cells, generally within sinusoids, with cytoplasmic light brown pigment (interpreted as pigment laden macrophages/Kupffer cells). There is one locally extensive zone with numerous clusters of similar pigment laden round cells in the hepatic parenchyma. There are rare clusters of leukocytes, sometimes consisting of mononuclear cells and other times consisting also of neutrophils, randomly scattered in the hepatic parenchyma. The portal zones in some portions of liver are subjectively slightly close together suggesting a depletion of intervening parenchyma. The individual hepatocytes are also subjectively small.

Diagnoses:

1. Capsular fibrosis and mild chronic mononuclear and mildly suppurative inflammation (suggestive of peritonitis)

2. Minimal mononuclear portal hepatitis

3. Mildly enhanced hepatocellular mitotic rate, presumptive

4. Minimal randomly scattered mononuclear and occasionally suppurative hepatitis

5. Locally extensive moderate accumulation of pigment laden macrophages/Kupffer cells

6. Minimal extra-medullary hematopoiesis

7. Subjective decreased distance between portal zones (suggesting acinar collapse/decreased hepatic parenchyma) and subjectively small hepatocytes.

Brain- There is fairly extensive dark neuron artifact (presumptive secondary to dissection).

Heart- There are rare individual cardiac myocytes with slightly more darkly eosinophilic cytoplasm than neighboring cells and with more homogenous and darkly eosinophilic chromatin staining in in contracted and shrunken nuclei.

Diagnoses:

1. Minimal individual myocytes change, interpreted as probable degerative change.

Kidney- The renal capsule is segmentally broadened with fibrous connective tissue that is occasionally infiltrated with small numbers of mononuclear leukocytes and neutrophils.

Diagnoses:

1. Capsular fibrosis and mild chronic mononuclear and mildly suppurative inflammation (suggestive of peritonitis)

Lung- The alveolar air spaces in some areas are mildly collapsed presumed secondary to dissection technique. There are rare megakaryocytes in the pulmonary parenchyma (EMH). There is scant alveolar hemorrhage presumed secondary to euthanasia. Neutrophils are in mildly enhanced numbers in alveolar air spaces and septal walls in a few scattered regions. The tip of the lung lobe has a focal accumulation of slightly increased numbers of foamy macrophages on alveolar septal walls and occasionally in alveoli. Uncommonly, pneumocytes lining alveolar septal walls have expanded cytoplasm (pneumocyte hypertrophy). There is one focus of perivascular lymphoid cuffing at the tip of one lung lobe.

Diagnoses:

1. Pneumonitis, mild, suppurative

2. Extramedullary hematopoiesis (EMH), mild

1213:

Liver- The liver capsule exhibits no significant histologic lesions. A few aggregates of mononuclear leukocytes and neutrophils are randomly scattered in the hepatic parenchyma. A few portal zones are infiltrated with modest numbers of mononuclear leukocytes including lymphocytes and plasma cells and presumptive macrophages. Rare neutrophils are sometimes also in affected portal zones. The staining properties of red blood cells in the section is faint and light brown material is noted in several sinusoids. This material is interpreted as faintly-staining red blood cells based on comparisons to other red blood cells in the section.

Diagnoses:

1. Minimal randomly scattered mononuclear and suppurative hepatitis

2. Minimal lymphoplasmacytic and histiocytic portal hepatitis.

3. Sinusoidal brown pigment accumulation interpreted as probable artifact of red blood cell staining.

Brain- There is fairly extensive dark neuron artifact interpreted as an artifact of dissection. There is extremely rare mild extravasation of blood into Virchow-Robbins’ space. The habenular nucei have a mesh-work of cells (presumptive neurons)with smudged nuclear features.

Diagnoses:

1. Locally extensive nuclear smudging in the habenular nuclei (a finding of uncertain significance).

2. Minimal extravasation of blood into Virchow-Robbins’ space.

Heart- There are rare individual cardiac myocytes with increased cytoplasmic eosinophilia and bland darkly staining contracted nuclei.

Diagnoses:

1. Minimal individual myocytes change, interpreted as probable degerative change.

Kidney- No significant histologic lesions are noted.

Lung- A few scattered alveoli are lined by pneumocytes with expansive cytoplasm (pneumocyte hypertrophy). There is scant acute hemorrhage in a few alveoli (attributed presumptively to euthanasia). Slightly enhanced numbers of neutrophils are occasionally in alveolar septal walls on in alveolar air spaces, particularly in instances of hypertrophied pneumocytes. Locally extensive areas of the lung parenchyma are collapsed, presumed secondary to dissection technique.

Diagnoses:

1. Minimal pneumocyte hypertrophy sometimes associated with minimally enhanced numbers of neutrophils.

1214:

Liver- The liver capsule is segmentally widened with fibrous connective tissue that is infiltrated with lymphocytes, plasma cells, macrophages, presumptive hemosiderin laden macrophages, and few neutrophils. There are occasional degenerate cells among the inflammatory infiltrates. There are rare accumulations of brown granular material in sinusoidal spaces, presumptively within Kupffer cells. A few portal zones and one central zone are infiltrated with few lymphoid cells, rare neutrophils, and occasional brown-pigment laden presumptive macrophages. There rare scattered mitotic hepatocytes. There are rare parenchymal clusters of presumptive hematopoietic precursors (EMH).

Diagnoses:

1. Capsular fibrosis and mild to moderate mononuclear inflammation including presumptive hemosiderin laden macrophages and few neutrophils(suggestive of peritonitis)

2. Locally extensive mimimal accumulation of brown pigment presumptively within Kupffer cells

3. Minimally enhanced hepatocellular mitotic rate, presumptive

4. Minimal extramedullary hematopoiesis

Brain- There is moderate dark neuron artifact interpreted as an artifact of dissection. There is one focus of white matter vacuolation in a segment of tissue that is at the edge of a torn piece of the tissue. Evaluation of deeper and more anatomically intact sections is required.

Diagnoses:

1. Focal white matter vacuolation of uncertain significance (deeper sections required).

Heart- There are rare individual cardiac myocytes with clear vacuoles within their cytoplasm.

Diagnoses:

1. Minimal presumptive cardiac myocyte vacuolation (suggestive of a degenerative change).

Kidney- The renal capsule is segmentally widened with fibrous connective tissue. There is a locally extensive region of renal cortex that extends from the capsule deep to deep cortex in which numerous tubules are filled with luminal degenerate cell debris including presumptive degenerative neutrophils. Many tubules in this region, both one with exudate and ones without, are smaller than nearby typical tubules and are lined by renal tubular epithelial cells with abundant basophilic cytoplasm (interpreted as regenerative). The renal interstitium in this zone is multifocally infiltrated with moderate numbers of lymphoid cells, neutrophils, and occasional pigment laden macrophages (interpreted as probable hemosiderin laden macrophages). The renal surface overlying the region of inflammation is moderately concave and there is a robust blood vessel in the region that is cuffed with moderate numbers of lymphoid cells. The renal medulla and deep cortex are not in section; the section only includes cortex of the kidney.

Diagnoses:

1. Pyelonephritis, locally extensive, suppurative, severe.

2. Capsular fibrosis (suggestive of peritonitis)

3. Incomplete sampling of the renal tissue (cortex only included in the section).

Lung- Neutrophils are moderately numerous percolating through alveolar septal walls and occasionally within alveolar air spaces. Occasional alveolar pneumocytes have expansive cytoplasm. The pulmonary parenchyma is multifocally collapsed, presumed secondary to dissection technique.

Diagnoses:

1. Pneumonitis, suppurative, moderate

1217:

Liver- The liver capsule is segmentally broadened by fibrous connective tissue that is multifocally to segmentally infiltrated with numerous lymphoid cells and sometimes with a few scattered macrophages with brown cytoplasmic material (hemosiderin presumptive) and occasional clusters of moderate numbers of neutrophils. There are some segments in which inflammatory cells, particularly pigment laden macrophages, and prominently along the interface between the hepatic parenchyma and the fibrotic capsule. A few to moderate numbers of scattered portal zones are infiltrated with moderately numerous lymphoid cells, rare neutrophils, and modest numbers of pigment laden macrophages. There are occasional central veins that have lymphoid infiltrates in modest abundance surrounding it as well as several scattered clusters of pigment laden macrophages. There are rare aggregates of few neutrophils and mononuclear leukocytes in the hepatic parenchyma. There is a locally extensive area in which there are scattered clusters of small numbers of presumptive Kupffer cells laden with abundant brown granular cytoplasmic pigment. Hepatocellular size is subjectively smaller than that observed in samples 1213 and 1214.

Diagnoses:

1. Capsular fibrosis and mild to moderate lymphocytic inflammation, occasional suppurative inflammation, and scattered pigment laden macrophages (suggestive of peritonitis)

2. Mild mononuclear and minimally suppurative portal hepatitis with occasional intralesional pigment laden macrophages

3. Mild peri-central lymphocytic hepatitis with accumulation of intra-lesional pigment laden macrophages.

4. Minimal randomly scattered mononuclear and occasionally suppurative hepatitis

5. Locally extensive moderate accumulation of pigment laden macrophages/Kupffer cells.

6. Subjectively smaller hepatocytes than in samples 1213 and 1214.

Brain- The cerebellum is not in section. There is extensive dark neuron artifact interpreted as an artifact of dissection.

Heart- There are are scattered individual cardiac myocytes with clear cytoplasmic vacuoles.

Diagnoses:

1. Minimal presumptive cardiac myocyte vacuolation (suggestive of a degenerative change).

Kidney- The renal sections are predominantly through cortex tissue with only minimal sampling of renal papilla. Very few lymphoid cells are occasionally in the connective tissue around robust blood vessels deep in the cortex (presumptively near the pelvis).

Lung- Scattered groups of alveoli have pneumocytes with moderately expansive cytoplasm lining their walls sometimes with enhanced numbers of neutrophils both within alveolar septal walls and in alveolar air spaces. There is one poorly sectioned presumptive megakaryocyte in the pulmonary parenchyma adjacent to a focal linear accumulation of granular amphophilic material.

Diagnoses:

1. Pneumonitis, suppurative, mild to moderate, with presumptive pneumocyte hypertrophy

1233:

Liver- There are rare to occasional individual degenerate hepatocytes sometimes with adjacent mononuclear cell infiltrates. Some degenerate hepatocytes are not associated with leukocyte infiltrates or other cell or tissue reaction. There are occasional mitotic hepatocytes. The hepatic capsule is non-remarkable. Occasional portal zones are infiltrated with modest numbers of lymphocytes and plasma cells and small numbers of neutrophils. There are rare clusters of lymphoid cells, histiocytes, and few neutrophils randomly scattered in the hepatic parenchyma.

Diagnoses:

1. Mildly enhanced hepatocellular mitotic rate

2. Scattered individual hepatocellular degeneration sometimes with presumptive lymphocytic infiltrates.

3. Mild mononuclear and minimally suppurative portal hepatitis

4. Minimal randomly scattered mononuclear and minimally suppurative hepatitis

Brain- There is abundant dark neuron artifact, presumed secondary to dissection. The habenular nuclei are minimally in section.

Heart- Histologic lesions are not discerned.

Kidney- One kidney section includes cortex, medulla, and papilla, while the second section includes only cortex. There is a single focus in the cortex in which there is a cluster of closely apposed small renal tubules lined by crowded plump cuboidal tubular epithelial cells with amphophilic to lightly basophilic cytoplasm (suggestive of a cluster of regenerative tubules). Scant degenerate cell debris is intermixed in this focus. There is a second minimal focus suggestive of but not clearly inactive of a similar change.

Diagnoses:

1. Focal presumptive renal tubular degeneration and regeneration.

Lung- Scattered regions of the pulmonary parenchyma are collapsed, presumed secondary to dissection technique. There rare scattered alveolar septal walls lined by pneumocytes with expansive cytoplasm (pneumocyte hypertrophy) sometimes with neutrophils associated with these expansive pneumocytes, in alveolar spaces, or within alveolar septal walls. There is a locally extensive region in which scattered alveolar septal walls are more prominently widened with modest numbers of mononuclear cells. There is a focal accumulation of several presumptive macrophages with abundant basophilic cytoplasm and few mixed mononuclear leukocytes and neutrophils in an alveolus.

Diagnoses:

1. Pneumonitis, mild, suppurative with presumptive hypertrophied pneumocytes

2. Focal histiocytic and mixed mononuclear and suppurative inflammation, minimal, focal.

1237:

Liver- The liver capsule is segmentally markedly broadened with fibrous connective tissue that is infiltrated by lymphoid cells and macrophages, including macrophages with brown granular cytoplasmic material, and rare neutrophils. Moderate numbers of aggregates of modest numbers of macrophages, lymphoid cells, and few neutrophils are randomly scattered in the hepatic parenchyma. Some of these inflammatory foci include a few brown pigment laden cells interpreted as macrophages. One inflammatory focus is more extensive and includes moderately abundant degenerate presumptive hepatocellular debris and numerous neutrophils among the inflammatory infiltrates. Moderately numerous scattered portal zones are infiltrated with moderate to slightly striking numbers of lymphoid cells, macrophages, and few neutrophils. A few central veins have aggregates of mononuclear leukocytes and few neutrophils adjacent to them. There are rare clusters of presumptive macrophages with brown granular cytoplasmic pigment scattered in the hepatic parenchyma. There are rare scattered individual degenerate hepatocytes. There are very rare clusters of hematopoietic precursor cells in the hepatic parenchyma. There are very rare mitotic hepatocytes.

Diagnoses:

1. Capsular fibrosis, marked, with lymphohistiocytic and minimally neutrophilic inflammatory infiltrates and pigment laden macrophages. (suggestive of peritonitis)

2. Mild to moderate randomly scattered mononuclear and occasionally suppurative hepatitis, rarely with intralesional degenerate hepatocytes.

3. Mild to moderate mononuclear and minimally neutrophilic portal hepatitis.

4. Mild peri-central lymphocytic and minimally neutrophilic hepatitis.

Mildly enhanced hepatocellular mitotic rate, presumptive

5. Rare pigment laden presumptive macrophages accumulation.

6. Rare scattered individual degenerate hepatocytes

7. Minimal extramedullary hematopoiesis.

8. Subjective decreased distance between portal zones (suggesting acinar collapse/decreased hepatic parenchyma) and subjectively small hepatocytes.

9. Minimally enhanced hepatocellular mitotic rate.

Brain- The brain sections are fragmented. Dark neuron artifact is present, presumptively secondary to dissection.

Heart- There are scattered moderately numerous individual cardiac myocytes with glassy slightly more darkly eosinophilic cytoplasm that surrounding cells and with homogenously staining darkly basophilic contracted nuclei.

Diagnoses:

1. Mild individual myocytes change, interpreted as probable degerative change.

Kidney- Very minimal numbers of lymphocytes infiltrate around a few small blood vessels near the renal pelvis. One kidney section includes cortex, medulla, and papilla, while the second is through cortex only. The renal capsule is segmentally minimally widened with fibrous connective tissue. There is one focus in which modest numbers of lymphocytes infiltrate the interstitium of the cortex.

Diagnoses:

1. Minimal lymphocytic interstitial nephritis

Lung- Neutrophils in small to modest numbers are in alveolar septal walls and also occasionally in alveolar air spaces. Occasional alveolar pneumocytes have expansive lightly eosinophilic cytoplasm.

Diagnoses:

1. Pneumonitis, mild, neutrophilic
